# Image Manipulation Detection via Multiscale Region and Cross-Image Edge Contrastive Learning

**DOI:** 10.3390/jimaging12100480

**Published:** 2026-10-02

**Authors:** Xiaoyi Zhou, Wenzhong Tang, Jinyu Zhang, Haoxiang Zhang, Bowei Wang, Shuai Wang

**Affiliations:** School of Computer Science and Engineering, Beihang University, Beijing 100191, China; zhouxiaoyi@buaa.edu.cn (X.Z.); tangwenzhong@buaa.edu.cn (W.T.); owtn66@163.com (J.Z.); zhang_haoxiang@buaa.edu.cn (H.Z.)

**Keywords:** image manipulation detection, pixel-level localization, contrastive learning, multiscale feature representation, cross-image edge modeling, digital image forensics

## Abstract

Pixel-level image manipulation localization requires separating weak forensic traces from image content while recovering manipulated regions under post-processing and domain shift. Existing methods do not jointly impose structured discrimination on multiscale region features and edge features drawn from different images. We therefore propose a dual contrastive learning framework built on a Mesorch-based convolutional–Transformer detector. Multiscale Region Contrastive Learning (MRCL) applies intra-image directional contrast to authentic and manipulated samples from four hierarchically fused feature scales. Cross-Image Edge Contrastive Learning (CECL) uses Canny-derived authentic edges and mask-derived manipulated boundary bands to construct within-image contrast, while separate historical queues provide manipulated positives and authentic negatives for cross-image contrast. Both objectives regularize training without changing the active prediction path at inference. Under Protocol-MVSS, models are trained on CASIAv2 and evaluated on COVERAGE, Columbia, NIST16, CASIAv1, and IMD2020. Across seeds 42, 43, and 44, the complete model improves mean F1 and IoU over the Mesorch-based binary-cross-entropy baseline on all five test sets. In the descriptive comparison with representative methods, its three-seed mean is highest for both metrics on NIST16 and IMD2020 and for F1 on CASIAv1, with the second-highest IoU on CASIAv1.

## 1. Introduction

Image editing and generative tools have made copy-move, splicing, object removal, and inpainting widely accessible. These operations alter local statistics, high-frequency residuals, boundary continuity, and contextual structure, but compression, resizing, blurring, and noise can suppress the resulting traces. Pixel-level manipulation localization must therefore recover the spatial support of weak forensic signals while remaining robust to image content and distribution shifts.

Passive forensic methods initially relied on handcrafted cues such as copy-move correspondences, keypoints, compression statistics, CFA traces, illumination inconsistency, and geometric constraints [1,2,3,4,5,6,7,8,9,10,11]. Learning-based methods subsequently introduced residual modeling, multiscale supervision, boundary-aware objectives, dual-stream feature extraction, and global context modeling, as represented by ManTra-Net [12], MVSS-Net [13], PSCC-Net [14], TruFor [15], IML-ViT [16], and Mesorch [17]. DiffIML [18] further approaches localization through score-based mask generation. These developments improve dense prediction, but discriminative detectors are still commonly trained primarily through pixel-level segmentation and architectural supervision. Such objectives do not directly organize authentic and manipulated samples at multiple semantic levels or use edge features from different images to constrain the representation space.

Contrastive approaches address parts of this problem from different perspectives. CFL-Net [19] and Proposal Contrastive Learning [20] construct local-region or proposal-level relationships, while NCL-IML [21] assigns contour patches non-mutually exclusive contrastive roles. CECL-Net [22] combines edge reconstruction with within-image foreground–background contrast; SAFIRE [23] uses source-aware contrast for source-region partitioning; and UnionFormer [24] emphasizes multiview interaction and boundary-sensitive supervision. These formulations target local regions, candidate proposals, contour roles, or source consistency. They do not combine intra-image region discrimination over a hierarchy of fused feature scales with memory-backed cross-image edge discrimination in a single localization framework.

We build such a framework on a Mesorch-based detector with convolutional and Transformer streams. Multiscale Region Contrastive Learning (MRCL) samples balanced authentic and manipulated features from four hierarchically fused stages and applies a weighted intra-scale directional contrastive objective. Cross-Image Edge Contrastive Learning (CECL) samples authentic image edges outside the manipulated neighborhood and manipulated features from a mask-derived boundary band. It combines bidirectional within-image edge contrast with a cross-image term that compares current manipulated features with historical manipulated positives and authentic negatives stored in separate circular queues. MRCL and CECL are auxiliary training objectives; the active prediction path at evaluation remains unchanged.

The main contributions of this work are summarized as follows.

- We formulate MRCL as four-scale intra-image region contrast over hierarchically fused convolutional–Transformer features, directly constraining authentic/manipulated separation at different semantic levels.
- We formulate CECL with distinct authentic-edge and manipulated-boundary samples, combining within-image contrast with historical cross-image relationships maintained by two class-specific memory banks.
- We evaluate the framework under Protocol-MVSS with three-seed component ablations, single-factor sensitivity analysis, controlled cost profiling, and comparison with representative methods. The complete model improves mean F1 and IoU over the Mesorch-based binary-cross-entropy baseline on all five test sets; the proposed method is strongest for both metrics on NIST16 and IMD2020 and achieves the highest F1 with the second-highest IoU on CASIAv1.

## 2. Related Work

### 2.1. Traditional Image Manipulation Detection Methods

Handcrafted forensic methods localize manipulation through operation- or acquisition-specific inconsistencies. Copy-move methods identify duplicated content through block matching [1], SIFT correspondences [2], or geometrically constrained keypoint configurations [3]. Compression and acquisition analyses use DCT statistics, double-JPEG artifacts, or CFA interpolation traces [4,5,6]. Splicing detectors instead examine statistical or camera-response inconsistency, transform-domain structure, phase congruency, and moment descriptors [7,8,9,10], while tampering possibility maps combine multiple cues for deletion and inpainting scenarios [11].

These methods connect a prediction to an interpretable forensic trace, but their features and decision rules are tied to particular manipulation processes, acquisition pipelines, or compression histories. Recompression, resizing, smoothing, and noise can weaken the assumed cue, and a cue designed for one manipulation type may not support dense localization of another. This limited transfer across operations and processing conditions motivated data-driven forensic representations.

### 2.2. Deep Learning-Based Manipulation Localization

Learning-based forensics first focused on separating manipulation traces from image semantics. High-pass filtering and constrained convolution direct CNNs toward residual evidence [25,26], while RGB-N combines visual content with a noise stream [27]. MFCN casts splicing localization as joint region and boundary prediction [28], and ManTra-Net treats manipulation traces as local anomalies learned through self-supervision and feature normalization [12]. These approaches established residual-sensitive dense prediction as an alternative to fixed forensic descriptors.

Subsequent architectures expanded the spatial and contextual scope of localization. BusterNet models source and target regions for copy-move detection, SPAN aggregates spatial context at multiple scales, and Constrained R-CNN integrates residual-sensitive filters into a detection framework [29,30,31]. PSCC-Net progressively refines masks across scales, MVSS-Net combines noise, boundary, and image-level supervision, and edge-aware message passing propagates information between region and boundary representations [13,14,32]. These methods strengthen dense supervision and spatial refinement without explicitly organizing region classes throughout the feature hierarchy.

Transformer and multiview models add long-range context to forensic traces. TBFormer uses two Transformer branches. TruFor combines RGB content with a noise-sensitive fingerprint, while Mesorch couples a convolutional stream for local traces with a Transformer stream for global context [15,17,33]. UnionFormer further integrates RGB, noise, and object-consistency views with boundary-sensitive interaction and contrastive supervision [24]. This line motivates the dual-stream representation used in our framework, while leaving the design of the region- and edge-level training objectives open.

Generative approaches provide a different formulation. DiffForensics introduces diffusion priors with edge-enhanced fine-tuning [34]. DiffIML models the mask distribution through latent score-based generation, using a mask-specific variational autoencoder, artifact-conditioned image encoding, a lightweight denoising network, edge supervision, and an error prior [18]. Rather than generating the mask iteratively, the present work retains direct discriminative prediction and regularizes its intermediate region and edge representations.

### 2.3. Contrastive Learning for Image Manipulation Detection

Region-level contrastive methods organize manipulation-related features beyond the final segmentation loss. CFL-Net contrasts local authentic and manipulated regions [19], Proposal Contrastive Learning forms positive and negative sets from candidate regions and multiview representations [20], and SAFIRE uses source-aware contrast to partition manipulated regions by origin [23]. Their contrastive units are local regions, proposals, or source partitions. MRCL instead applies one region-separation objective independently to four hierarchically fused feature stages, allowing shallow and deep representations to receive the same class-structured supervision.

Edge-oriented contrastive methods target the transition between authentic and manipulated content. NCL-IML assigns contour patches different contrastive roles through a dual-branch pivot design [21], while CECL-Net combines edge reconstruction with within-image foreground–background contrast [22]. UnionFormer couples boundary-sensitive multiview interaction with contrastive supervision [24]. These objectives operate on samples from the current image or its source partition. CECL extends the sampling scope with historical edge features: current manipulated-boundary queries use manipulated history as positives and authentic-edge history as negatives.

The proposed framework connects these two levels without conflating them. MRCL supplies four weighted intra-image, intra-stage region losses; CECL supplies within-image edge contrast and memory-backed cross-image contrast. Their joint role is to organize the intermediate representation used by a direct pixel-level localizer, rather than to add another prediction branch.

## 3. Proposed Methodology

### 3.1. Overview

**Figure 1:**
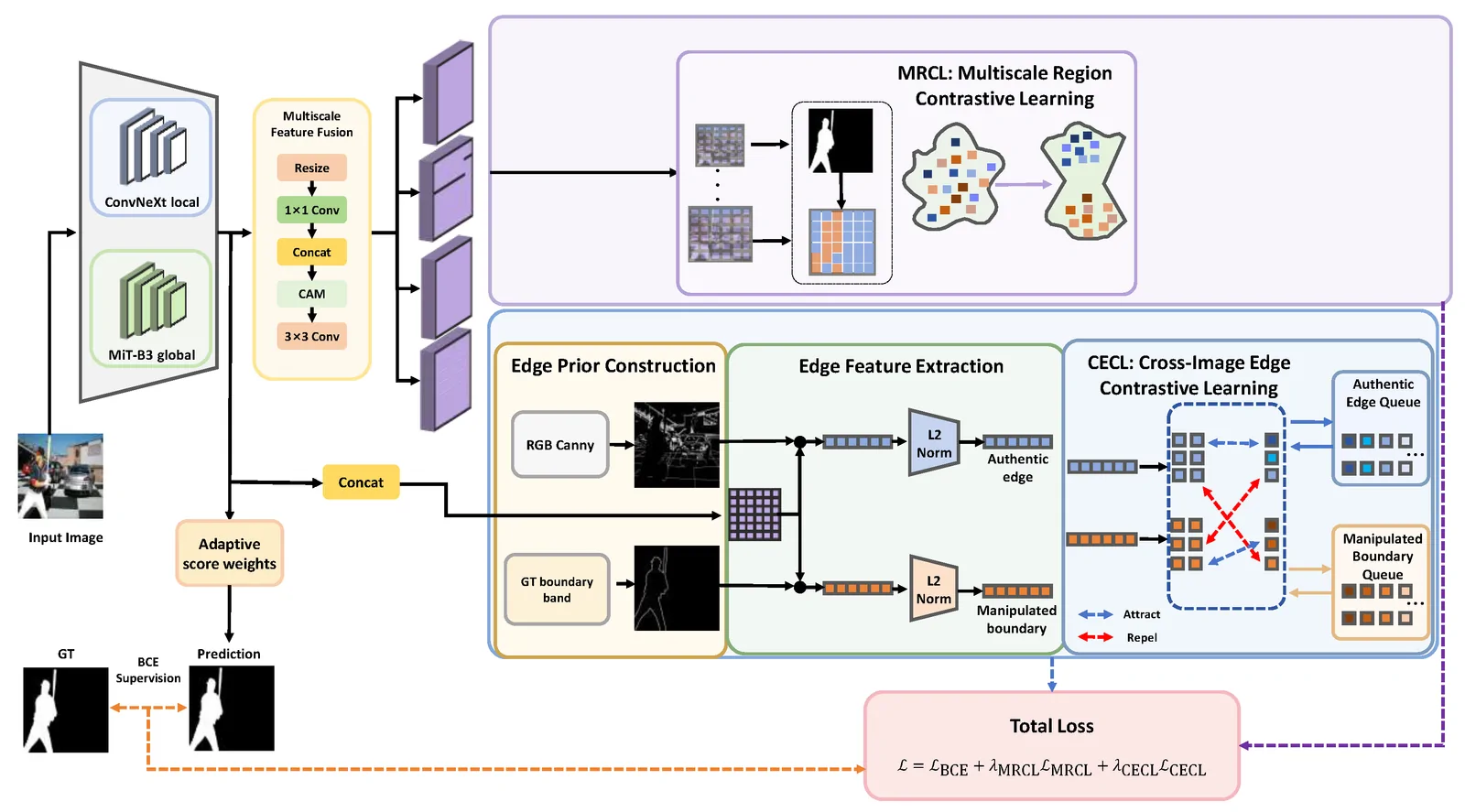
Overall architecture of the proposed framework. A convolutional stream extracts local forensic evidence, and a Transformer stream models global context. MRCL operates on four pairwise fused stage features, while CECL operates on the concatenated multistage feature with authentic-edge and manipulated-boundary samples. The two contrastive objectives are used during training. Blue- and orange-toned squares schematically denote authentic and manipulated feature samples, respectively.

Given an input image $X_{i}$, the model predicts a pixel-level manipulation mask ${\hat{Y}}_{i}$ supervised by $Y_{i}\in {\left\{0,1\right\}}^{H_{0}\times W_{0}}$. The framework contains a Mesorch-based dual-stream prediction path and two auxiliary training objectives. MRCL applies region contrast to four hierarchically fused feature maps, whereas CECL applies within- and cross-image contrast to authentic-edge and manipulated-boundary features. Figure 1 shows how the prediction and contrastive paths share the backbone representations.

The convolutional stream is ConvNeXt-Tiny with stage depths (3, 3, 9, 3) and channel dimensions (96, 192, 384, 768); its input concatenates RGB with a high-frequency DCT reconstruction. The Transformer stream is SegFormer MiT-B3 with stage depths (3, 4, 18, 3), attention heads (1, 2, 5, 8), and channel dimensions (64, 128, 320, 512); its input concatenates RGB with a low-frequency DCT reconstruction. ConvNeXt supplies local residual-sensitive features, while MiT-B3 supplies long-range contextual features at corresponding hierarchical stages.

For image *i* in a mini-batch of size *B*, let ${\left\{F_{i,l}^{c}\right\}}_{l=1}^{L}$ and ${\left\{F_{i,l}^{t}\right\}}_{l=1}^{L}$ denote the convolutional and Transformer features, respectively. We use L=4 stages at 1/4, 1/8, 1/16, and 1/32 of the input resolution. Each stage feature is upsampled to the 1/4 grid, and the two aligned stream features at stage *l* are fused as

(1) $$F_{i,l}^{f}=F_{\mathrm{fusion}}\left(F_{i,l}^{c},F_{i,l}^{t}\right),\;l=1,\ldots ,L.$$

The fusion operator first projects each stream to 128 channels with a 1×1 convolution–LayerNorm–ReLU block. The projected maps are concatenated and recalibrated by a channel-attention branch comprising global average pooling, two 1×1 convolutions with an intermediate ReLU, and a sigmoid gate. A 3×3 convolution–LayerNorm–ReLU block then reconstructs a 128-channel output. Consequently, $F_{i,l}^{f}\in R^{128\times H_{0}/4\times W_{0}/4}$ preserves the semantic stage of its inputs on a shared spatial grid.

The three paths consume these representations differently. The prediction path projects the eight upsampled backbone maps to one-channel logits. A score network receives the nine-channel concatenation of RGB, high-frequency DCT, and low-frequency DCT inputs and predicts eight softmax weights; the weighted logit sum is bilinearly resized to the input resolution. MRCL uses the four 128-channel maps in Equation (1). CECL directly concatenates all eight upsampled backbone maps into a 640-channel feature. At inference, only the prediction path is active.

### 3.2. Multiscale Region Contrastive Learning

**Figure 2:**
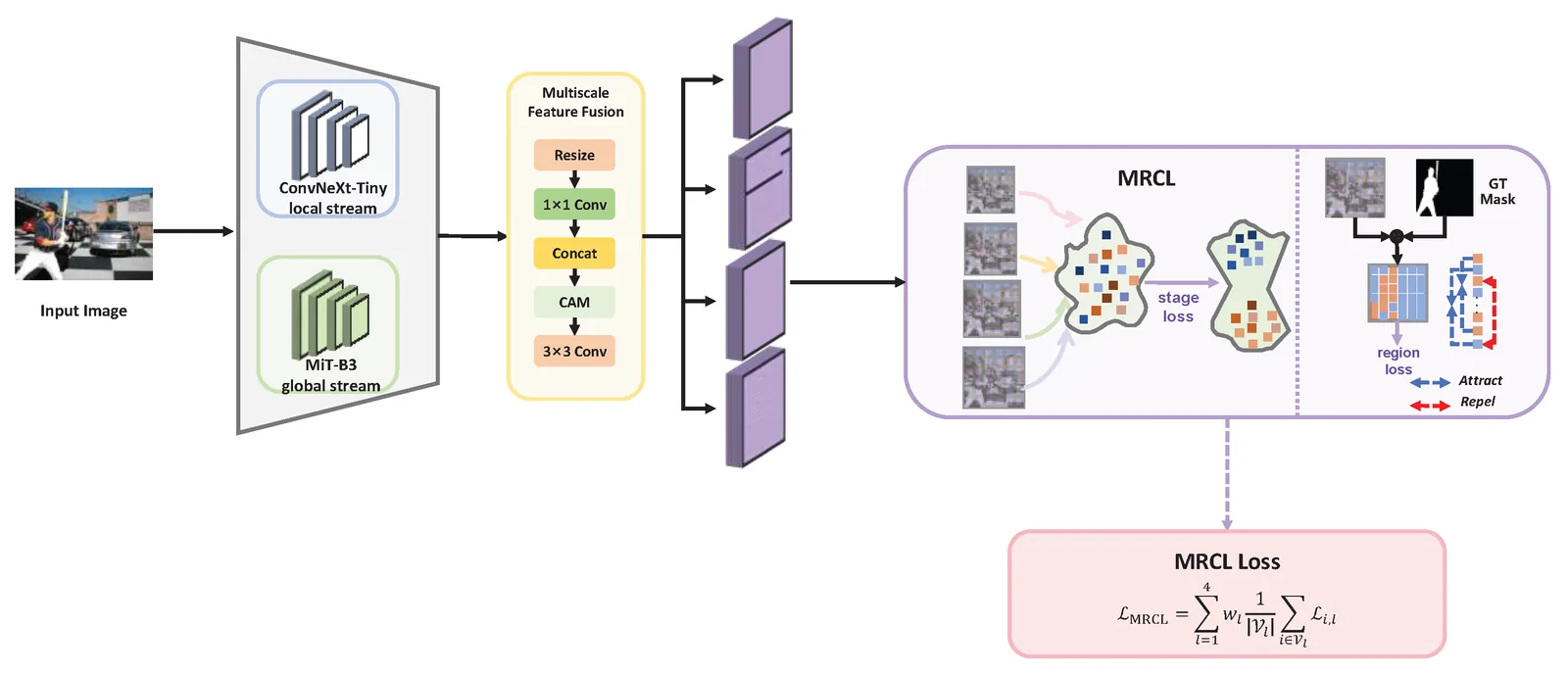
Multiscale Region Contrastive Learning (MRCL). Authentic and manipulated features are sampled from each hierarchically fused stage. Blue- and orange-toned squares denote authentic and manipulated region feature samples, respectively. A directional contrastive loss is computed within each image and stage, and the four stage losses are combined by fixed weights.

Binary mask supervision constrains the final prediction but does not directly organize authentic and manipulated samples in intermediate feature space. A single stage also cannot represent local residuals and contextual inconsistency at the same semantic level. MRCL therefore applies region contrast independently to each of the four fused stages and combines the resulting losses with stage-specific weights. Figure 2 illustrates the four-stage feature sampling and region contrastive objective.

#### 3.2.1. Region Feature Sampling

For fused feature map $F_{i,l}^{f}$, the ground-truth mask is resized to the same spatial grid as $Y_{i,l}\in {\left\{0,1\right\}}^{H_{l}\times W_{l}}$. A location is authentic when $Y_{i,l}\left(u,v\right)=0$ and manipulated when $Y_{i,l}\left(u,v\right)=1$. With $f_{i,l,u,v}^{f}\in R^{C_{l}}$ denoting the feature at (u,v), the two region sets are

(2) $$A_{i,l}=\left\{f_{i,l,u,v}^{f}|Y_{i,l}\left(u,v\right)=0\right\},\;T_{i,l}=\left\{f_{i,l,u,v}^{f}|Y_{i,l}\left(u,v\right)=1\right\}.$$

At most 1000 features are randomly sampled from each class, after which both samples are truncated to the same cardinality. We denote the balanced sets by ${\tilde{A}}_{i,l}$ and ${\tilde{T}}_{i,l}$. An image–stage pair is valid when both sets contain at least two features; all other pairs are skipped. The sampled features are $\ell_{2}$-normalized before cosine similarity is computed.

#### 3.2.2. Multiscale Region Contrastive Objective

MRCL uses a directional intra-stage objective: authentic features provide the queries and positive pool, and manipulated features provide the negatives. For query set $Q={\left\{q_{j}\right\}}_{j=1}^{m}$ and negative set *N*, the mean non-self positive logit for $q_{j}$ is

(3) $$p_{j}\left(Q\right)=\frac{1}{m-1}\sum_{\begin{array}{c} k=1\\ k\neq j \end{array}}^{m}\mathrm{sim}\left(q_{j},q_{k}\right),$$

where sim(·,·) denotes cosine similarity. The directional contrastive loss is

(4) $$L_{\mathrm{dir}}\left(Q,N;\tau \right)=-\frac{1}{m}\sum_{j=1}^{m}\log\frac{\exp\left(p_{j}\left(Q\right)/\tau \right)}{\exp\left(p_{j}\left(Q\right)/\tau \right)+\sum_{n\in N}\exp\left(\mathrm{sim}(q_{j},n)/\tau \right)},$$

where τ is the temperature. The region loss for valid image *i* at stage *l* is

(5) $$L_{i,l}^{r}=L_{\mathrm{dir}}\left({\tilde{A}}_{i,l},{\tilde{T}}_{i,l};\tau_{r}\right).$$

Let $V_{l}$ contain the valid images at stage *l*. MRCL averages their losses within each stage and then weights the four stages:

(6) $$L_{\mathrm{MRCL}}=\sum_{l=1}^{4}w_{l}\frac{1}{|V_{l}|}\sum_{i\in V_{l}}L_{i,l}^{r}.$$

An empty $V_{l}$ contributes zero. We use $\tau_{r}=0.1$. From the shallowest to the deepest stage, the weights are $w_{1}=0.20$, $w_{2}=0.25$, $w_{3}=0.35$, and $w_{4}=0.55$. The weighted sum exposes local residual-sensitive and contextual features to the same region-separation objective while retaining stage-specific representations.

### 3.3. Cross-Image Edge Contrastive Learning

**Figure 3:**
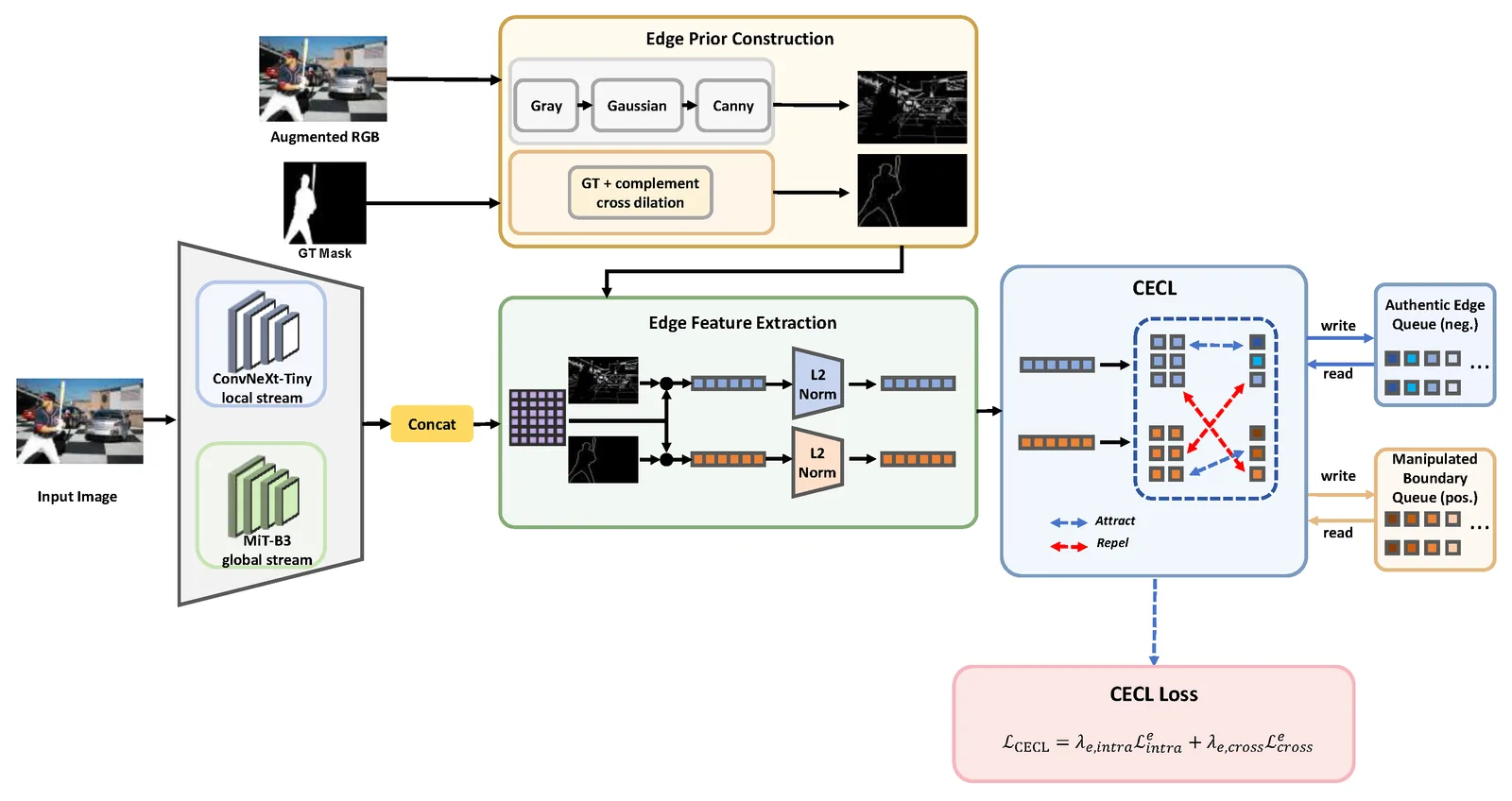
Cross-Image Edge Contrastive Learning (CECL). Authentic-edge features are sampled from Canny edges outside the manipulated neighborhood, and manipulated-boundary features are sampled from a mask-derived band. Blue- and orange-toned squares denote authentic-edge and manipulated-boundary feature samples, respectively. Separate queues provide historical features for cross-image contrast.

MRCL structures region features but does not explicitly distinguish authentic image edges from manipulated boundary transitions. Because boundary samples occupy a small part of each image, within-image contrast also provides a limited sample distribution. CECL addresses both points by combining balanced within-image edge contrast with historical cross-image contrast. Figure 3 illustrates the edge-prior construction, feature extraction, and use of historical queues.

#### 3.3.1. Edge Feature Sampling

For image *i*, CECL directly concatenates the eight upsampled backbone maps into $F_{i}^{e}\in R^{640\times H_{e}\times W_{e}}$; it does not use an additional projection head. Edge priors are generated after data augmentation and before final resizing and padding. The priors undergo the same subsequent transform as the ground-truth mask and are resized to the grid of $F_{i}^{e}$ by nearest-neighbor interpolation.

The authentic-edge prior retains Canny edges away from the manipulated region. The augmented RGB image is converted to grayscale, smoothed with a 5×5 Gaussian filter at σ=2, and processed with Canny thresholds 50 and 150. A one-step dilation of the mask with a 5×5 square excludes the manipulated neighborhood:

(7) $$E_{i,\mathrm{auth}}={\mathrm{Canny}}_{50,150}\;\left(G_{5,2}\left(\mathrm{Gray}\left(X_{i}\right)\right)\right)⊙\left[1-{\mathrm{Dilate}}_{□,5}\left(Y_{i}\right)\right].$$

The manipulated-boundary prior is the overlap between one-step dilations of the mask and its complement using a 3×3 cross-shaped structuring element:

(8) $$E_{i,\mathrm{tam}}={\mathrm{Dilate}}_{\times ,3}\left(Y_{i}\right)⊙{\mathrm{Dilate}}_{\times ,3}\left(1-Y_{i}\right).$$

This fixed one-step operation defines the band directly; no separate radius parameter is used. It selects features on both sides of the transition between manipulated and authentic regions.

Let $f_{i,u,v}^{e}\in R^{640}$ denote the CECL feature at (u,v). The authentic-edge and manipulated-boundary feature sets are

(9) $$A_{i}^{e}=\left\{f_{i,u,v}^{e}|E_{i,\mathrm{auth}}\left(u,v\right)=1\right\},\;T_{i}^{e}=\left\{f_{i,u,v}^{e}|E_{i,\mathrm{tam}}\left(u,v\right)=1\right\}.$$

At most 1024 features are randomly sampled from each set and truncated to a shared cardinality. The balanced authentic-edge and manipulated-boundary samples are denoted by $Z_{i}^{a}$ and $Z_{i}^{t}$, respectively. A sample pair is valid when both sets contain at least two features; all other pairs are skipped. Features are $\ell_{2}$-normalized for the loss and before queue insertion.

#### 3.3.2. Dual Memory Bank

CECL maintains separate historical queues for the two feature classes:

(10) $$M_{a}\in R^{K\times D_{e}},\;M_{t}\in R^{K\times D_{e}},$$

Here, $M_{a}$ stores authentic-edge features and $M_{t}$ stores manipulated-boundary features. Both queues use K=4096, $D_{e}=640$, and FP32 storage. Each training rank maintains an independent pair of circular queues without momentum updates or cross-rank gathering. For every valid image, CECL reads the historical entries to compute the loss and then enqueues the current normalized features. Each pointer advances circularly and overwrites older entries after wrap-around.

#### 3.3.3. Edge Contrastive Objective

Within an image, each class alternates as the directional query and positive pool while the other class supplies negatives. Using Equation (4), the loss for valid image *i* is

(11) $$L_{i,\mathrm{intra}}^{e}=\frac{1}{2}L_{\mathrm{dir}}\left(Z_{i}^{t},Z_{i}^{a};\tau_{e}\right)+\frac{1}{2}L_{\mathrm{dir}}\left(Z_{i}^{a},Z_{i}^{t};\tau_{e}\right).$$

For cross-image contrast, $m_{i}$ is the minimum cardinality of the current manipulated-boundary set and the two valid historical queues. We retain $m_{i}$ current queries ${\tilde{Z}}_{i}^{t}\subseteq Z_{i}^{t}$ and randomly sample equally sized historical sets ${\tilde{M}}_{t}\subseteq M_{t}$ and ${\tilde{M}}_{a}\subseteq M_{a}$. Query ${\tilde{z}}_{i,j}^{t}$ is paired with historical manipulated-boundary positive ${\tilde{m}}_{i,j}^{t}$, and every feature in ${\tilde{M}}_{a}$ acts as an authentic-edge negative:

(12) $$L_{i,\mathrm{cross}}^{e}=-\frac{1}{m_{i}}\sum_{j=1}^{m_{i}}\log\frac{\exp\left(\mathrm{sim}\left({\tilde{z}}_{i,j}^{t},{\tilde{m}}_{i,j}^{t}\right)/\tau_{e}\right)}{\exp\left(\mathrm{sim}\left({\tilde{z}}_{i,j}^{t},{\tilde{m}}_{i,j}^{t}\right)/\tau_{e}\right)+\sum_{n\in {\tilde{M}}_{a}}\exp\left(\mathrm{sim}\left({\tilde{z}}_{i,j}^{t},n\right)/\tau_{e}\right)}.$$

The mini-batch averages of the valid within- and cross-image terms are denoted by $L_{\mathrm{intra}}^{e}$ and $L_{\mathrm{cross}}^{e}$. CECL combines them as

(13) $$L_{\mathrm{CECL}}=\lambda_{e,\mathrm{intra}}L_{\mathrm{intra}}^{e}+\lambda_{e,\mathrm{cross}}L_{\mathrm{cross}}^{e},$$

where $\lambda_{e,\mathrm{intra}}=0.6$, $\lambda_{e,\mathrm{cross}}=0.4$, and $\tau_{e}=0.1$.

### 3.4. Overall Training Objective

Training combines binary cross-entropy on the predicted mask with the MRCL and CECL objectives:

(14) $$L=L_{\mathrm{BCE}}+\lambda_{\mathrm{MRCL}}L_{\mathrm{MRCL}}+\lambda_{\mathrm{CECL}}L_{\mathrm{CECL}},$$

where $\lambda_{\mathrm{MRCL}}=0.3$ and $\lambda_{\mathrm{CECL}}=0.2$. The BCE term supervises the output mask, MRCL structures authentic and manipulated region features at four stages, and CECL structures authentic-edge and manipulated-boundary features within and across images. The contrastive terms are removed with their auxiliary paths at inference.

## 4. Experiments and Results

### 4.1. Experimental Settings

#### 4.1.1. Datasets

We follow the Protocol-MVSS setting in IMDL-BenCo [35]: CASIAv2 [36] is used for training, and COVERAGE [37], Columbia [8], NIST16 [38,39], CASIAv1 [36], and IMD2020 [40] are used for evaluation without further fine-tuning. The dataset manifests contain 5123 CASIAv2 training pairs and 100, 180, 564, 920, and 2010 test pairs for the five test sets, respectively.

COVERAGE emphasizes copy-move manipulation in self-similar content, whereas Columbia primarily contains splicing with relatively clear transitions. NIST16 and IMD2020 include more heterogeneous editing pipelines and stronger post-processing. These differences allow the protocol to test localization across distinct manipulation and image characteristics.

CASIAv1 and the CASIAv2 training set belong to the same dataset family. The manifests used here contain no exact image or mask matches between them, but their shared provenance makes CASIAv1 a related-dataset transfer test. The other four test sets provide evaluation on independent dataset sources.

#### 4.1.2. Evaluation Metrics

Pixel-level F1 and IoU are used as the evaluation metrics. Manipulated pixels are treated as the positive class, and probability maps are thresholded at 0.5 to obtain binary predictions. Both metrics are computed for each image and then macro-averaged over the test set. F1 balances precision and recall for manipulated-pixel detection, whereas IoU measures the spatial overlap between the predicted and ground-truth masks.

#### 4.1.3. Implementation Details and Comparison Protocol

Inputs are processed at 512×512. Training uses four GPUs with a batch size of 10 per rank and two-step gradient accumulation, yielding an effective batch size of 80. AdamW is applied for 200 epochs with an initial learning rate of $1\times 10^{-4}$, a minimum learning rate of $5\times 10^{-7}$, weight decay of 0.05, and cosine decay after two warmup epochs.

The controlled study includes the Mesorch-based Baseline, two single-objective variants, and the Full model. The Baseline uses the same dual-stream prediction architecture with BCE supervision but excludes MRCL and CECL; the two intermediate variants add either MRCL or CECL, and Full includes both objectives. All four variants are trained on the CASIAv2 manifest with seeds 42, 43, and 44. This CASIAv2-only protocol differs from the official Mesorch seven-source balanced training recipe, so the Baseline provides a controlled architectural reference for the proposed objectives.

The Transformer and convolutional streams are initialized from pretrained MiT-B3 and convnexttiny.in12kftin1k checkpoints, respectively. In both six-channel input stems, pretrained RGB weights initialize the first three channels, and the added frequency channels use seeded Kaiming initialization. Experiments use Python 3.8.10, PyTorch 2.0.1 with CUDA 11.7, torchvision 0.15.2, and timm 0.9.12 on NVIDIA RTX 3090 GPUs (NVIDIA Corporation, Santa Clara, CA, USA).

The default MRCL configuration uses $\tau_{r}=0.1$, at most 1000 samples per class, and stage weights (0.20, 0.25, 0.35, 0.55). CECL uses $\tau_{e}=0.1$, at most 1024 samples per class, two per-rank 4096×640 queues, and intra-/cross-image weights of 0.6/0.4. The outer loss weights are $\lambda_{\mathrm{MRCL}}=0.3$ and $\lambda_{\mathrm{CECL}}=0.2$.

All external-method results other than DiffIML are seed-42 values reported in Tables 3 and 12 of IMDL-BenCo under Protocol-MVSS [35]. The DiffIML row uses our Protocol-MVSS reproduction with seed 42 [18]. The cross-method Ours row reports the mean over seeds 42, 43, and 44, while the internal ablation tables report the mean and standard deviation across the same three runs.

#### 4.1.4. Computational Cost

Table 1 compares the Baseline and Full model on an NVIDIA RTX 3090 using FP32, batch size 1, 512×512 inputs, 30 warm-up iterations, and 100 measured iterations. Measurements cover the complete forward pass, including DCT preprocessing, and exclude data loading, metric computation, and image export.

The training-only branches add 1.333 M total parameters (1.55%), while both models retain 84.983 M active evaluation parameters and 121.532 counted GMACs. Their latency differs by less than 1.3%, and throughput and peak inference memory are also nearly unchanged. Thus, MRCL and CECL do not materially alter the active inference path. The operation count includes convolution, linear, matrix multiplication, layer normalization, and bilinear upsampling; unsupported nonlinear and element-wise operations are excluded.

Training cost increases because both contrastive objectives are active. In the seed-42 run, the Baseline and Full models require 125.046 and 187.103 s per epoch, corresponding to a 1.50× increase. Their per-rank peak allocated memory is 16.415 and 19.969 GiB, and peak reserved memory is 17.633 and 21.846 GiB, corresponding to 1.22× and 1.24× increases. The two FP32 queues additionally occupy 20 MiB per rank during training and are removed at inference.

### 4.2. Comparison with Representative Methods

The comparison includes ManTra-Net [12], MVSS-Net [13], CAT-Net [41], ObjectFormer [42], PSCC-Net [14], NCL-IML [21], TruFor [15], IML-ViT [16], and DiffIML [18]. Together, they represent local forensic modeling, multiscale and boundary-aware learning, Transformer context modeling, contrastive localization, and score-based mask generation. Table 2 and Table 3 report their pixel-level F1 and IoU results under Protocol-MVSS.

The proposed method ranks first in both metrics on NIST16 and IMD2020. On NIST16, its F1 and IoU reach 0.376 and 0.312, compared with the best alternative values of 0.331 and 0.276. On IMD2020, it reaches 0.425/0.356, followed by DiffIML at 0.408/0.348. On CASIAv1, it ranks first in F1 and second in IoU.

The ranking changes on COVERAGE and Columbia, where IML-ViT and TruFor, respectively, obtain the strongest results. The proposed method records 0.348/0.286 on COVERAGE and 0.731/0.696 on Columbia, showing that cross-dataset performance varies with manipulation characteristics. The rankings are descriptive because Ours averages three seeds, whereas every external row reports a seed-42 result.

### 4.3. Ablation Study

The ablation isolates the two objectives on the same Mesorch-based prediction architecture. Baseline uses BCE supervision alone; the next two variants add MRCL or CECL individually, and Full combines them. Table 4 and Table 5 report mean ± standard deviation over seeds 42, 43, and 44.

Each objective improves the three-seed mean F1 and IoU over Baseline on all five datasets. MRCL contributes the larger individual gain on NIST16 and CASIAv1, whereas CECL contributes the larger gain on COVERAGE, Columbia, and IMD2020. Full achieves the highest mean for every dataset and metric; relative to Baseline, its F1 gains range from 0.0419 to 0.1267 and its IoU gains range from 0.0398 to 0.1291. The consistent combined improvement supports the complementary roles of region- and edge-level contrast. The reported standard deviations quantify across-seed variability rather than between-method significance.

### 4.4. Hyperparameter Sensitivity Analysis

Seven single-factor variants test the loss weights, temperatures, and queue capacity around the Default setting ($\lambda_{\mathrm{MRCL}}=0.3$, $\lambda_{\mathrm{CECL}}=0.2$, $\tau_{r}=\tau_{e}=0.1$, and K=4096). Table 6 and Table 7 report the per-dataset results and five-dataset means. These runs use seed 42 and 200 epochs and are analyzed separately from the three-seed ablation.

Default gives the highest five-dataset mean F1 and IoU, at 0.5231 and 0.4691. Larger MRCL and CECL weights retain similar mean F1-values but reduce mean IoU, while increasing either temperature or reducing the queue capacity lowers both aggregate metrics.

Individual datasets favor different variants: a lower MRCL weight performs best on NIST16, a higher MRCL weight performs best on Columbia and IMD2020, and the CECL-weight variants give the strongest COVERAGE results. Default retains the best CASIAv1 scores and the strongest aggregate performance, making it the most balanced tested configuration. Because this study uses one seed per setting, the sensitivity results describe parameter behavior rather than statistical significance.

### 4.5. Qualitative Results and Failure Cases

**Figure 4:**
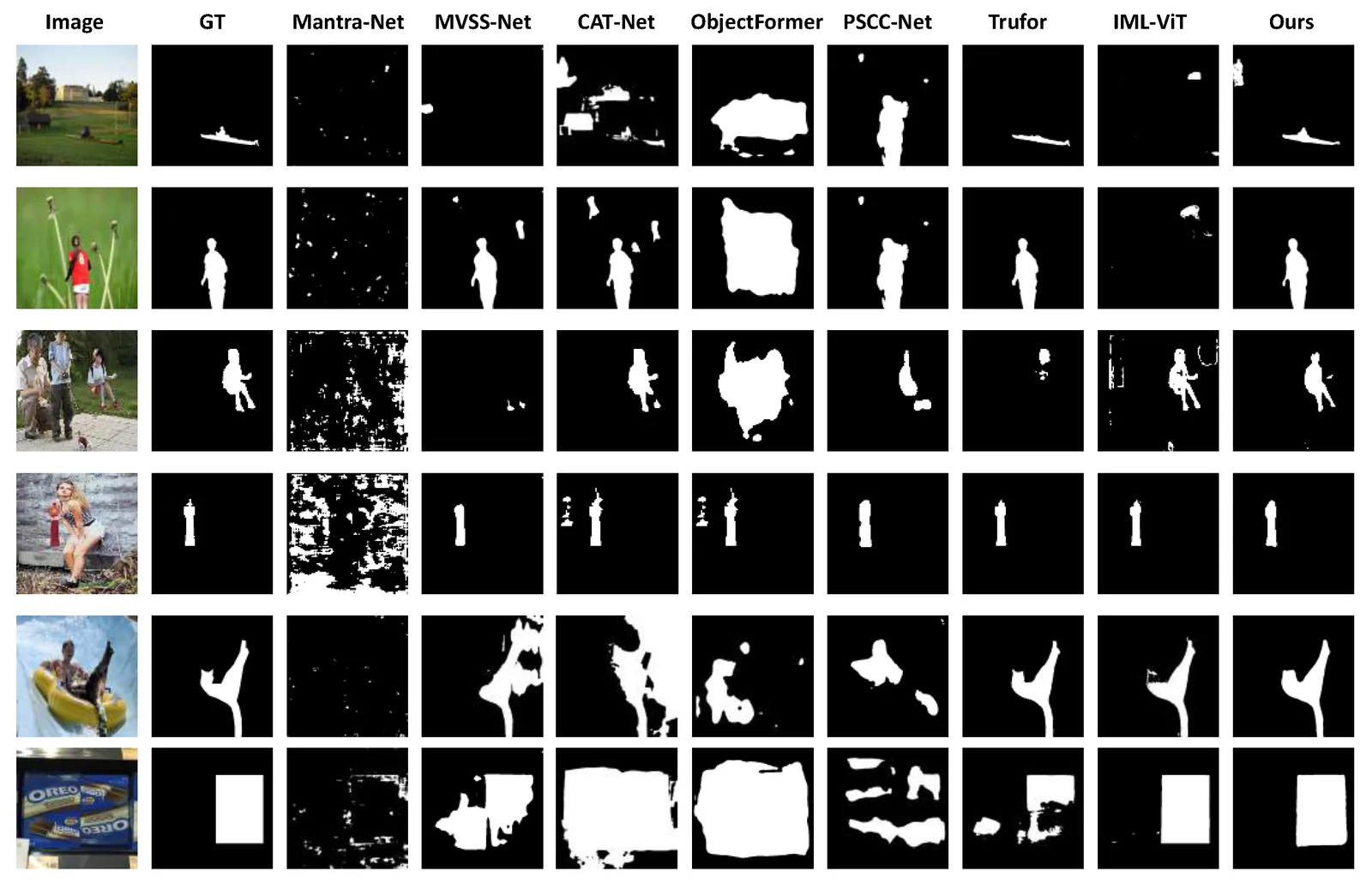
Qualitative comparison of manipulation localization results. From left to right, the columns show the input image, ground-truth mask, predictions of ManTra-Net, MVSS-Net, CAT-Net, ObjectFormer, PSCC-Net, TruFor, IML-ViT, and our method. White pixels indicate predicted manipulated regions.

Figure 4 compares localization masks for small objects, human-shaped regions, slender structures, irregular silhouettes, and rectangular pasted regions. Across these cases, competing predictions exhibit three recurring errors: scattered background activation, over-expanded regions, and fragmented or incomplete masks.

The proposed method generally preserves the extent of slender and human-shaped regions while reducing background activation around irregular and rectangular manipulations. Its displayed masks are compact and their boundaries are more continuous in these examples. This evidence is qualitative; Table 2 and Table 3 provide the region-level quantitative comparison.

**Figure 5:**
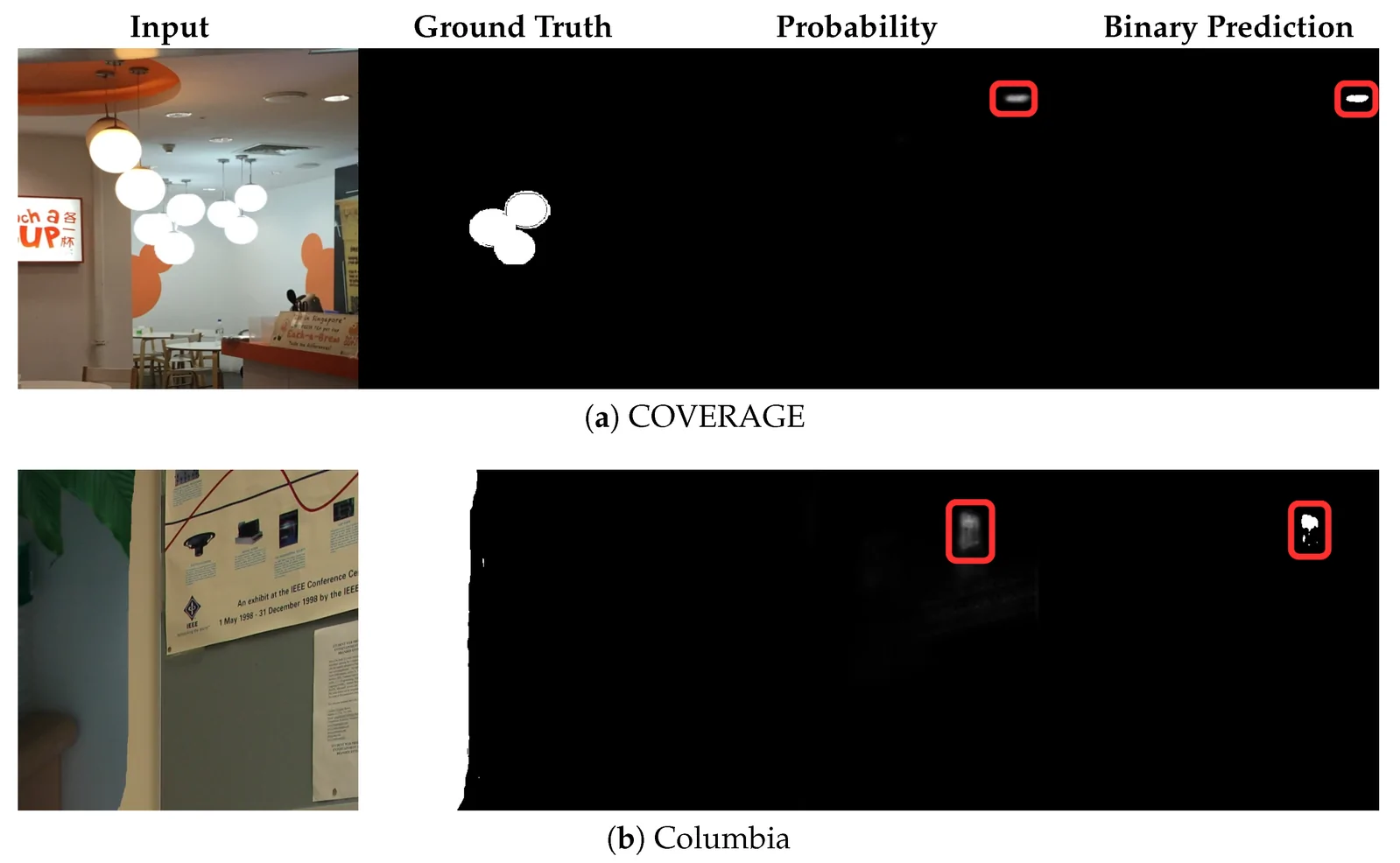
Deterministically selected failure cases from COVERAGE and Columbia. For each dataset, the sample with the lowest pixel-level F1-score is selected, with ties broken by sample identifier. The probability maps are thresholded at 0.5 to obtain the binary predictions. Red boxes highlight the small false-positive responses. Both examples yield pixel-level F1 = 0 and IoU = 0.

Figure 5 presents deterministically selected failure cases from COVERAGE and Columbia, two datasets on which the model is relatively less competitive. For each dataset, we select the sample with the lowest pixel-level F1-score, breaking ties by sample identifier.

Both cases are dominated by false negatives: the annotated manipulated regions are almost entirely missed, while the model responds only to small incorrect areas. These examples reveal a failure mode under cross-dataset transfer in which the manipulation cues in the target region are insufficiently preserved for reliable localization.

## 5. Discussion

The experiments show that the benefit of the proposed framework comes from combining two feature-space objectives with pixel-level supervision. Across three seeds, the Full model improves mean F1 and IoU over the Mesorch-based baseline on every test set. The cross-method results are more varied, identifying datasets on which the proposed representation is effective and settings in which other localization paradigms remain stronger.

### 5.1. Interpretation of Cross-Dataset Results

The proposed method records the highest F1 and IoU among the compared methods on NIST16 and IMD2020. These datasets contain heterogeneous editing operations and post-processing, so the result indicates that joint multiscale region discrimination and cross-image edge discrimination transfer beyond the CASIAv2 training distribution. On COVERAGE and Columbia, however, the method trails the strongest alternatives. The weaker COVERAGE result points to self-similar copy-move manipulation as a remaining challenge, while the Columbia result shows that the proposed objectives do not supersede strong forensic representations in every splicing setting.

On CASIAv1, the method obtains the highest F1 and the second-highest IoU. CASIAv1 and the CASIAv2 training set are releases from the same dataset family. Although the manifests used here contain no exact image or mask matches, their common provenance makes CASIAv1 a related-dataset transfer test rather than independent cross-domain evidence. The NIST16, IMD2020, COVERAGE, and Columbia results carry the cross-dataset interpretation in this evaluation.

### 5.2. Relation to Previous Studies

Previous localization methods improve generalization through residual priors, multiscale supervision, boundary-aware learning, or Transformer context modeling [13,14,15,17]. The proposed framework operates at the objective level: MRCL organizes authentic and manipulated region features at four semantic scales, and CECL organizes authentic-edge and manipulated-boundary features within and across images. This makes the two objectives compatible with the local-forensic and global-context representations already supplied by the dual-stream backbone.

The comparison also shows that no single modeling paradigm dominates every dataset. TruFor is strongest on Columbia, IML-ViT is strongest on COVERAGE, and DiffIML is second on Columbia and closest to the proposed method on IMD2020. The proposed method is strongest on NIST16 and IMD2020 and near the lead on CASIAv1. Thus, score-based mask generation, forensic priors, global context modeling, and contrastive feature regularization retain different dataset-dependent strengths under Protocol-MVSS.

### 5.3. Effectiveness of MRCL and CECL

The three-seed ablation identifies a complementary pattern. MRCL provides larger mean gains than CECL on NIST16 and CASIAv1, whereas CECL provides larger gains on COVERAGE, Columbia, and IMD2020. Combining the objectives yields the highest mean F1 and IoU on every dataset in the ablation, rather than exchanging performance between the two groups of test sets.

The sensitivity results refine this conclusion. The Default setting has the highest five-dataset mean F1 and IoU, at 0.5231 and 0.4691, while several alternative weights improve individual datasets. In particular, the preferred MRCL weight differs across NIST16, Columbia, and IMD2020, and the CECL-weight variants are strongest on COVERAGE. The default configuration is therefore a balanced setting across datasets rather than a universal per-dataset optimum. Because these variants use one seed, they characterize parameter behavior but not its across-seed significance.

Pixel F1 and IoU measure manipulated-region detection and overlap. They show that CECL improves the localization mask, but they do not isolate boundary displacement or boundary-specific accuracy. The more continuous boundaries visible in Figure 4 remain qualitative evidence.

### 5.4. Implications, Limitations, and Future Work

The results support treating feature-space discrimination as a complement to dense segmentation supervision. MRCL and CECL are used only during training, and the Full model retains the Baseline’s 84.983 M active evaluation parameters and 121.532 counted GMACs. This inference-time equivalence comes with a training cost: the controlled Full run requires 1.50× the Baseline’s epoch time and 1.22× its peak allocated memory per rank.

The method and evaluation also define clear limits. The lowest-F1 COVERAGE and Columbia examples miss most of the annotated region and respond to a small incorrect area. CECL depends on fixed Canny edges and a mask-derived boundary band, while its class-specific queues add training memory and computation. The quantitative evaluation covers pixel F1 and IoU rather than dedicated boundary metrics; the sensitivity study uses one seed; and the external comparison combines single-seed external-method rows with the three-seed mean of the proposed method. CASIAv1 further represents related-dataset transfer rather than an independent source.

Future work should prioritize source–target correspondence for self-similar copy-move cases, adaptive edge priors, and more efficient cross-image sampling. A matched multi-seed protocol with dedicated boundary metrics would also separate region-level gains from boundary-specific effects more directly, while broader data sources are needed to evaluate open-world and generative manipulations.

## 6. Conclusions

This work introduced two complementary training objectives for a Mesorch-based dual-stream manipulation localizer. MRCL applies directional region contrast to balanced authentic and manipulated samples from four fused feature scales. CECL combines within-image contrast between authentic-edge and manipulated-boundary features with cross-image contrast supported by class-specific historical queues. These objectives regularize region and edge representations without changing the active prediction path at inference.

Across three seeds, the Full model improves mean F1 and IoU over the binary-cross-entropy baseline on all five Protocol-MVSS test sets. In the descriptive cross-method comparison, it achieves the highest F1 and IoU on NIST16 and IMD2020 and the highest F1 with second-highest IoU on CASIAv1, while trailing the strongest methods on COVERAGE and Columbia. The ablation and sensitivity results show complementary module effects and a balanced default configuration across datasets. The current evidence supports region-level localization gains; boundary behavior is assessed qualitatively because the quantitative evaluation is limited to pixel F1 and IoU. Together, these results support multiscale region contrast and memory-backed cross-image edge contrast as complementary training objectives, with future work needed on self-similar copy-move cases, boundary-specific evaluation, and training efficiency.

## Figures and Tables

**Table 1:** Controlled Baseline/Full inference cost. Parameter entries are reported as total/active-at-evaluation, and operation entries as counted GMACs/GFLOPs under the 2 FLOPs per MAC convention.

Model	Parameters (M)	GMACs/GFLOPs	Latency (ms)	Throughput (img/s)	Peak Memory (GB)
Baseline	85.754/84.983	121.532/243.064	33.291	30.04	3.161
Full	87.087/84.983	121.532/243.064	32.844	30.45	3.167

**Table 2:** Pixel-level F1 under Protocol-MVSS. Ours is the mean over seeds 42, 43, and 44. The best and second-best results are highlighted in bold and underlined.

Method	COVERAGE	Columbia	NIST16	CASIAv1	IMD2020
ManTra-Net	0.090	0.243	0.104	0.125	0.055
MVSS-Net	0.259	0.386	0.246	0.534	0.279
CAT-Net	0.296	0.584	0.269	0.581	0.273
ObjectFormer	0.294	0.336	0.173	0.429	0.173
PSCC-Net	0.231	0.604	0.214	0.378	0.235
NCL-IML	0.225	0.446	0.260	0.502	0.237
TruFor	0.419	**0.865**	0.324	0.721	0.322
IML-ViT	**0.435**	0.780	0.331	0.721	0.327
DiffIML	0.382	0.821	0.329	0.719	0.408
Ours	0.348	0.731	**0.376**	**0.725**	**0.425**

**Table 3:** Pixel-level IoU under Protocol-MVSS. Ours is the mean over seeds 42, 43, and 44. Methods without reported benchmark IoU results are omitted. The best and second-best results are highlighted in bold and underlined.

Method	COVERAGE	Columbia	NIST16	CASIAv1	IMD2020
MVSS-Net	0.192	0.290	0.176	0.440	0.201
CAT-Net	0.204	0.469	0.196	0.509	0.198
ObjectFormer	0.181	0.224	0.111	0.320	0.106
PSCC-Net	0.146	0.477	0.150	0.310	0.157
TruFor	0.352	**0.832**	0.271	**0.666**	0.267
IML-ViT	**0.372**	0.685	0.250	0.648	0.264
DiffIML	0.332	0.787	0.276	0.656	0.348
Ours	0.286	0.696	**0.312**	0.661	**0.356**

**Table 4:** Pixel-level F1 ablation results under the Protocol-MVSS setting. Entries are mean ± standard deviation over seeds 42, 43, and 44. The baseline denotes the dual-stream detector trained only with pixel-level supervision. The best mean for each dataset is highlighted in bold.

Method	COVERAGE	Columbia	NIST16	CASIAv1	IMD2020
Baseline	0.2339±0.0214	0.6038±0.0487	0.3156±0.0128	0.6452±0.0286	0.3827±0.0162
Baseline + MRCL	0.2686±0.0232	0.6618±0.0564	0.3747±0.0052	0.7167±0.0155	0.4036±0.0069
Baseline + CECL	0.2955±0.0125	0.6984±0.0272	0.3417±0.0175	0.6809±0.0161	0.4159±0.0055
Full	0.3475±0.0275	0.7305±0.0282	0.3756±0.0208	0.7249±0.0143	0.4246±0.0220

**Table 5:** Pixel-level IoU ablation results under the Protocol-MVSS setting. Entries are mean ± standard deviation over seeds 42, 43, and 44. The baseline denotes the dual-stream detector trained only with pixel-level supervision. The best mean for each dataset is highlighted in bold.

Method	COVERAGE	Columbia	NIST16	CASIAv1	IMD2020
Baseline	0.1932±0.0189	0.5667±0.0455	0.2515±0.0116	0.5689±0.0301	0.3165±0.0147
Baseline + MRCL	0.2249±0.0224	0.6267±0.0517	0.3068±0.0037	0.6515±0.0174	0.3374±0.0073
Baseline + CECL	0.2400±0.0027	0.6596±0.0320	0.2737±0.0166	0.6126±0.0155	0.3432±0.0110
Full	0.2857±0.0204	0.6958±0.0256	0.3117±0.0157	0.6612±0.0187	0.3563±0.0195

**Table 6:** Pixel-level F1 sensitivity results for the proposed model. Default denotes the hyperparameter configuration used in the full proposed model. Each row is a seed-42, 200-epoch single-factor experiment. The best result in each column is highlighted in bold.

Setting	COVERAGE	Columbia	NIST16	CASIAv1	IMD2020	Mean
Default	0.3246	0.7622	0.3529	**0.7413**	0.4347	**0.5231**
$\lambda_{\mathrm{MRCL}}=0.15$	0.3005	0.6659	**0.4064**	0.6957	0.4235	0.4984
$\lambda_{\mathrm{MRCL}}=0.45$	0.3063	**0.7800**	0.3688	0.7071	**0.4520**	0.5228
$\lambda_{\mathrm{CECL}}=0.1$	**0.3588**	0.7098	0.3785	0.6935	0.4443	0.5170
$\lambda_{\mathrm{CECL}}=0.3$	0.3274	0.7693	0.3736	0.7025	0.4347	0.5215
$\tau_{r}=0.2$	0.2802	0.7662	0.3585	0.6999	0.4188	0.5047
$\tau_{e}=0.2$	0.3254	0.7600	0.3456	0.7071	0.4272	0.5131
K=2048	0.3332	0.7239	0.3796	0.6977	0.4491	0.5167

**Table 7:** Pixel-level IoU sensitivity results for the proposed model. Default denotes the hyperparameter configuration used in the full proposed model. Each row is a seed-42, 200-epoch single-factor experiment. The best result in each column is highlighted in bold.

Setting	COVERAGE	Columbia	NIST16	CASIAv1	IMD2020	Mean
Default	0.2753	0.7241	0.2957	**0.6828**	0.3679	**0.4691**
$\lambda_{\mathrm{MRCL}}=0.15$	0.2475	0.6278	**0.3351**	0.6221	0.3520	0.4369
$\lambda_{\mathrm{MRCL}}=0.45$	0.2472	**0.7398**	0.3012	0.6364	**0.3750**	0.4599
$\lambda_{\mathrm{CECL}}=0.1$	0.2761	0.6662	0.2981	0.6094	0.3596	0.4419
$\lambda_{\mathrm{CECL}}=0.3$	**0.2762**	0.7336	0.3033	0.6344	0.3634	0.4622
$\tau_{r}=0.2$	0.2330	0.7375	0.2950	0.6377	0.3513	0.4509
$\tau_{e}=0.2$	0.2692	0.7276	0.2787	0.6406	0.3546	0.4541
K=2048	0.2755	0.6886	0.3062	0.6266	0.3729	0.4540

## Data Availability

Access information for the third-party datasets used in this study is provided below, subject to the providers’ conditions. CASIAv1 and CASIAv2 are described in [36]; their localization masks are available at https://github.com/namtpham/casia1groundtruth and https://github.com/namtpham/casia2groundtruth. COVERAGE [37] is available at https://github.com/wenbihan/coverage; Columbia [8] at https://www.ee.columbia.edu/ln/dvmm/downloads/authsplcuncmp/; NIST16 [38,39] at https://doi.org/10.18434/mds2-2410 and https://mfc.nist.gov; and IMD2020 [40] at https://staff.utia.cas.cz/novozada/db/ (all links accessed on 1 October 2026). The experimental results supporting this study are included in the article. No new dataset was created. The source code is not publicly available at present due to restrictions associated with an ongoing research project.

## Abbreviations

The following abbreviations are used in this manuscript:

MRCL	Multiscale Region Contrastive Learning
CECL	Cross-Image Edge Contrastive Learning
BCE	Binary Cross-Entropy
CNN	Convolutional Neural Network
CUDA	Compute Unified Device Architecture
DCT	Discrete Cosine Transform
CFA	Color Filter Array
SIFT	Scale-Invariant Feature Transform
RGB	Red, Green, and Blue
JPEG	Joint Photographic Experts Group
IoU	Intersection over Union
F1	F1-score
FP32	32-bit Floating-Point Precision
GMACs	Giga Multiply–Accumulate Operations
GFLOPs	Giga Floating-Point Operations
GPU	Graphics Processing Unit
